# Effect of assisted hatching on pregnancy outcomes: a systematic review and meta-analysis of randomized controlled trials

**DOI:** 10.1038/srep31228

**Published:** 2016-08-09

**Authors:** Da Li, Da-Lei Yang, Jing An, Jiao Jiao, Yi-Ming Zhou, Qi-Jun Wu, Xiu-Xia Wang

**Affiliations:** 1 Center of Reproductive Medicine, Department of Obstetrics and Gynecology, Shengjing Hospital of China Medical University, Shenyang 110004, China; 2 Department of Medicine, Brigham and Women's Hospital, Harvard Institutes of Medicine, Harvard Medical School, Boston, MA 02115, USA; 3 Department of Clinical Epidemiology, Shengjing Hospital of China Medical University, Shenyang 110004, China

## Abstract

Emerging evidence suggests that assisted hatching (AH) techniques may improve clinical pregnancy rates, particularly in poor prognosis patients; however, there still remains considerable uncertainty. We conducted a meta-analysis to verify the effect of AH on pregnancy outcomes. We searched for related studies published in PubMed, Web of Science, and Cochrane library databases from start dates to October 10, 2015. Totally, 36 randomized controlled trials with 6459 participants were included. Summary odds ratios (ORs) with 95% confidence intervals (CIs) for whether by AH or not were estimated. We found a significant increase in clinical pregnancy (OR = 1.16, 95% CI = 1.00–1.36, *I*^2^ = 48.3%) and multiple pregnancy rates (OR = 1.50, 95% CI = 1.11–2.01, *I*^2^ = 44.0%) with AH when compared to the control. Numerous subgroup analyses stratified by hatching method, conception mode, extent of AH, embryos transfer status, and previous failure history were also carried out. Interestingly, significant results of clinical pregnancy as well as multiple pregnancy rates were observed among women who received intracytoplasmic sperm injection, and who received AH which the zona were completely removed. In summary, this meta-analysis supports that AH was associated with an increased chance of achieving clinical pregnancy and multiple pregnancy. Whether AH significantly changes live birth and miscarriage rates needs further investigations.

Assisted hatching (AH) techniques are the manipulation of the zona pellucida by laser, mechanical, or chemical means, with the aim of facilitating embryo implantation^1^. An emerging body of evidence suggests that AH may improve clinical pregnancy rates, particularly in poor prognosis patients^2^; however, there still remains considerable uncertainty. For example, two previous systematic reviews and meta-analyses have showed that AH does appear to offer a significantly increased chance of achieving a clinical pregnancy, especially in women with previous repeated failure or frozen-thawed embryos^3^,^4^. However, whether AH significantly improves the success rates of other several important outcomes, such as live birth and multiple pregnancy, or whether it is associated with negative consequences, such as miscarriage rates, has been still unsolved. Additionally, several limitations existed in previous two meta-analyses. For example, Carney *et al*.^3^ used the fixed-effect model to report their findings. This assumes that there is one identical true treatment effect common to every study, whereas the random-effect model assumes that the true treatment effect in any of the analysed studies may be different in each case. Notably, these two meta-analyses used different risk estimates and included different studies. The conclusions of these studies might be interpreted with caution. Herein, to further clarify the effect of assisted hatching on pregnancy outcomes, we updated the evidence from two previous meta-analyses by not only unifying the inclusion criteria as well as these included studies risk estimates, but also by including studies which were published in the recent five years.

## Results

### Search results, study characteristics, and quality assessment

**Figure 1:**
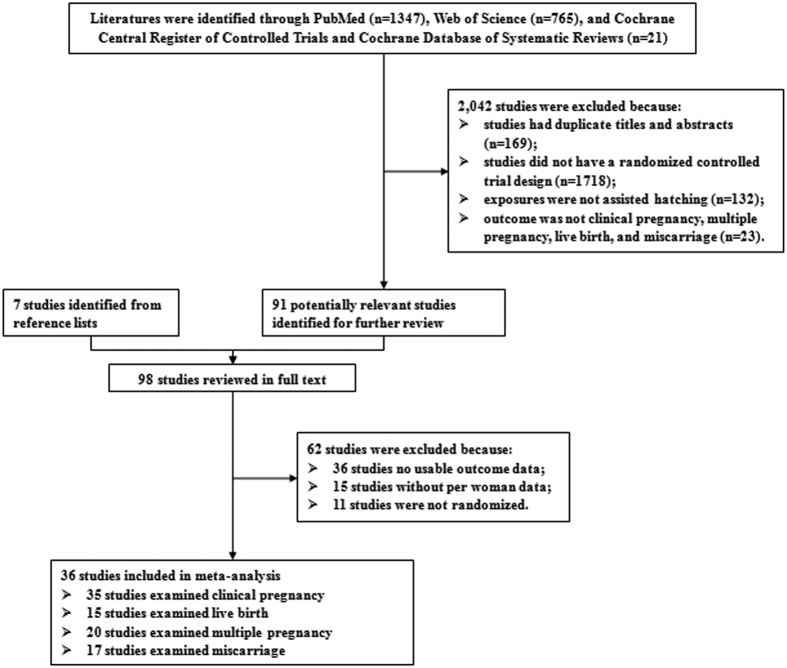
Flow-chart of study selection.

The detailed procedures of the literature search and screening are shown in Fig. 1. In brief, we retrieved 2,133 unique articles: 1,347 from PubMed, 765 from Web of Science, and 21 from Cochrane library databases. After application of our inclusion and exclusion criteria, 36 randomized controlled trials (RCTs) with a total of 6,459 participants were identified.

The data extracted from each included study are listed in Table 1. These studies were published between 1992 and 2014. Of these studies, eight studies were conducted in the USA^5^,^6^,^7^,^8^,^9^,^10^,^11^,^12^, five studies each in China^13^,^14^,^15^,^16^,^17^ and Turkey^18^,^19^,^20^,^21^,^22^, three studies each in Brail^23^,^24^,^25^ and Italy^26^,^27^,^28^, two studies each in Iran^29^,^30^ and Israel^31^,^32^, and one study each in Canada^33^, Germany^34^, Egypt^35^, Switzerland^36^, the Czech Republic^37^, Japan^38^, Australia^39^, and Belgium^40^. Women received either *in vitro* fertilisation (IVF) or intracytoplasmic sperm injection (ICSI) were observed in fourteen, nine, and seven studies, respectively. Additionally, thirty studies included transferred fresh embryos to women, and four included and frozen-thawed embryos. Sixteen studies included participants with a history of previous failure.

Supplementary Table S1 and Supplementary Figure S1 present the summaries of risk of bias for all the included studies. Except for the category of method for allocation, 24 studies (66.7%) had a low risk of bias; an unclear risk of bias accounted for the majority of the other categories.

### Clinical pregnancy

Thirty-six RCTs investigated the effect of AH on clinical pregnancy. Compared with those women in the control group, women who underwent AH was associated with a significant increase in clinical pregnancy rate (OR = 1.16, 95% CI = 1.00–1.36), with moderate heterogeneity (*I*^2^ = 48.3%) (see Supplementary Fig. S2). There was no evidence of publication bias (*P* = 0.93 for Egger’s test and *P* = 0.52 for Begg’s test). Although numerous subgroup analyses were carried out, not all of them revealed statistically significant results (see Table 2). For example, when stratified by hatching method, significant results were observed in chemical (OR = 1.26) and mechanical (OR = 1.68) methods. Additionally, we also observed significant results among women who had only received ICSI, who received AH which were completely removal of zona, who were transferred fresh embryos with a failure history, and who were transferred frozen-thawed embryos without a failure history. A sensitivity analysis omitting one study at a time and calculating the summarized ORs for the remainder of the studies showed that the 36 study-specific ORs ranged from a low of 1.13 (95% CI = 0.96–1.33; *I*^2^ = 46.1%) after omitting the study by Balaban *et al*.^19^, to a high of 1.21 (95% CI = 1.05–1.41; *I*^2^ = 35.5%) after omitting the study by Valojerdi *et al*.^30^.

### Live birth

Fifteen RCTs investigated the effect of AH on live birth. Compared with those women in the control group, women who underwent AH had a non-significant OR of live birth (OR = 1.09, 95% CI = 0.92–1.30), without heterogeneity (*I*^2^ = 0%) (see Supplementary Fig. S3). There was no evidence of publication bias (*P* = 0.31 for Egger’s test and *P* = 0.14 for Begg’s test). Similar non-significant results were consistent in these subgroup analyses (see Table 3). The 15 study-specific ORs ranged from a low of 1.05 (95% CI = 0.88–1.25; *I*^2^ = 0%) after omitting the study by Wan *et al*.^13^, to a high of 1.12 (95% CI = 0.94–1.33; *I*^2^ = 0%) after omitting the study by Balakier *et al*.^33^ in the sensitivity analyses.

### Multiple pregnancy

Twenty RCTs investigated the effect of AH on multiple pregnancy. Compared with those women in the control group, women who underwent AH was associated with a significant increase in multiple pregnancy (OR = 1.50, 95% CI = 1.11–2.01), with moderate heterogeneity (*I*^2^ = 44.0%) (see Supplementary Fig. S4). There was no evidence of publication bias (*P* = 0.65 for Egger’s test and *P* = 0.82 for Begg’s test). Among stratified analyses, we observed significant results in studies using the laser AH method among women who only received ICSI, who received AH which were completely removal of zona, who were transferred to fresh embryos, who did not have a previous failure history, and who were transferred fresh embryos without a failure history (see Table 4). The 20 study-specific ORs ranged from a low of 1.37 (95% CI = 1.04–1.79; *I*^2^ = 30.5%) after omitting the study by Balaban *et al*.^19^ to a high of 1.62 (95% CI = 1.23–2.13; *I*^2^ = 30.9%) after omitting the study by Valojerdi *et al*.^30^.

### Miscarriage

Seventeen RCTs investigated the effect of AH on miscarriage. Compared with those women in the control group, women who underwent AH had a non-significant OR of miscarriage (OR = 1.03, 95% CI = 0.72–1.48), without heterogeneity (*I*^2^ = 0%) (see Supplementary Fig. S5). There was no evidence of publication bias (*P* = 0.59 for Egger’s test and *P* = 0.54 for Begg’s test). Similar non-significant results were consistent in these subgroup analyses (see Table 5). The 17 study-specific ORs ranged from a low of 0.97 (95% CI = 0.66–1.42; *I*^2^ = 0%) after omitting the study by Wan *et al*.^13^ to a high of 1.09 (95% CI = 0.76–1.56; *I*^2^ = 0%) after omitting the study by Primi *et al*.^36^ in the sensitivity analyses.

## Discussion

This most up-to-date meta-analysis, including 36 RCTs with 6,459 participants, suggested that women who underwent AH was associated with a significant increase in clinical pregnancy and multiple pregnancy rate. Notably, significant results of clinical pregnancy as well as multiple pregnancy rates were observed among women who received ICSI, and who received AH which the zona were completely removed. However, non-significant results were observed in live birth and miscarriage rates in women who underwent AH compared with those in the control group.

Recently, several technologies of AH have been developed, including mechanical, chemical, laser and piezon. Although various methods of AH are available, previous studies suggested little difference in outcomes due to method^4^,^41^. Nevertheless, our study found women who underwent chemical or mechanical AH was associated with a significant increase in clinical pregnancy. In contrast, women who underwent laser AH was associated with a significant increase in multiple pregnancy rate. Compared with other methods, laser AH is the most popular and ideal technology, with following advantages: (i) it saves time and decreases the number of laser shots; (ii) embryos are outside the incubator for less time; (iii) the risk of temperature increase in the immediate vicinity of the embryos from laser thermal shock is minimized^13^. However, a potential problem with laser AH is heating of embryo cells near the breach site in the zona pellucida^42^. The local heating depends on the beam power and laser pulse duration^42^. On the other hand, the benefit of AH either opening or thinning the zona pellucida is still controversial. Previous studies reported that zona opening of mouse embryos might have adverse effects as (i) the possibility of loss of blastomeres or of the whole embryo during contractions of the female reproductive tract^43^ or (ii) the inhibition of natural expansion of blastocyst^44^. Furthermore, cruciate thinning of the human zona pellucida rather then a complete zona drilling was also shown to increase (i) blastocyst hatching^45^ and (ii) implantation rates^46^,^47^. Furthermore, previous studies mentioned that the quality of the embryo was a factor that can affect the outcome^48^. Our study presented that women who underwent AH with fresh embryos was associated with a significant increase in multiple pregnancy rate which was partly in line with the previous finding. Although positive point estimates were observed in outcome of clinical pregnancy and live birth, neither of them showed statistical significance. Therefore, further studies are warranted to confirm our findings as well as to investigate the AH effect on other outcomes.

A major strength of this meta-analysis was compliance with the PRISMA guidelines (Supplementary Table S2), and this meta-analysis lie in the number of RCT studies included latest studies and increased the statistical power to detect the effect of AH on several important outcomes. Our study generally concurs with and further reinforces the results of previous meta-analyses. Notably, numerous subgroup and sensitivity analyses were carried out to explore the heterogeneity, as well as to test the robustness of the findings. Additionally, both models (fixed and random effect) were used in this study according to the heterogeneity (instead of using either of them, as in the previous studies), which could best demonstrate the effect of AH on different outcomes.

Several limitations of this study also should be acknowledged. Firstly, compared to neonatal development or foetal malformations, the investigated outcomes of this study were short term. However, limited included studies evaluated these aforementioned long-term outcomes, which might be attributed to the RCTs’ design. More studies are warranted to investigate the effect of AH on long-term outcomes. On the other hand, publication bias can be a problem in the meta-analyses of published studies; however, we found no statistical evidence of publication bias in this study by Egger’s linear regression and Begg’s rank correlation methods, and there did not seem to be asymmetry in the funnel plots when inspected visually (data not shown).

In conclusion, based on the current meta-analysis, AH was associated with an increased chance of achieving clinical pregnancy and multiple pregnancy. Notably, significant results of clinical pregnancy as well as multiple pregnancy rates were observed among women who received intracytoplasmic sperm injection, and who received AH which the zona were completely removed. These findings were partly consistent with the recommendation of the American Society of Reproductive Medicine which suggested that individual assisted reproductive technology programmes should evaluate their own unique patient populations in order to determine which subgroups may benefit from AH. Notably, patients receiving AH should be selected with more scrupulosity recently. More studies, especially high quality RCTs, are needed to investigate the effect of AH on live birth, miscarriage, and other long-term outcomes.

## Methods

### Databases and search strategies

Two investigators (DL and Q-JW) systematically and independently searched the PubMed, Web of Science and Cochrane library databases from each database’s inception to the end of October 2015 for epidemiological studies, without restriction. The following search phrase was used: (zona pellucida OR assisted hatching) AND (implantation OR pregnancy OR live birth OR miscarriage). We also hand-screened references of relevant review articles to identify other potential studies. This study was carried out using a predetermined protocol in accordance with the Preferred Reporting Items for Systematic Reviews and Meta-analyses (PRISMA) reporting guidelines^49^ (Supplementary Table S2).

### Study selection and exclusion

Original studies were eligible if they: (i) had an randomized controlled trial (RCT) study design; (ii) evaluated the effect of AH human embryos compared with a control group in which embryos were not submitted to AH; (iii) the primary analysis was per woman randomized; and (iv) presented the data necessary for calculating the odds ratios (ORs) or relative risks (RRs) and 95% confidence intervals (CIs)^50^. Original studies were ineligible if they: (i) were observational studies, reviews without original data, ecological studies, editorials, or case reports; (ii) did not report any of the evaluated outcomes; (iii) invalid analysis (for example ’per cycle’ data); or (iv) did not report the data necessary for calculating the aforementioned risk estimates. Similar to our previous studies^50^,^51^,^52^,^53^,^54^,^55^,^56^,^57^, if there were several publications from the same study, we included the study with the most cases and relevant information.

### Data extraction and quality assessment

These investigators (DL, D-LY, JA and Q-JW) independently extracted the data of these included studies. A reviewer (DL) was involved to resolve all disagreements. From each eligible study, these investigators abstracted information independently on the primary author, year of publication, geographic location, age of intervention/control populations, conception mode, AH method, embryo transfer status, and whether participants had a previous failure history. In situations when only the number of populations of fourfold table were given, we calculated the estimate and 95% CI.

To determine the validity of these included trials, we assessed the risk of bias as advised by the Cochrane Collaboration^58^, including the domains of adequacy of randomization, allocation concealment, blinding, completion of outcome data, and selective reporting. If one or more domains were judged as being high or unclear, we classified the trial as having a high risk of bias.

### Statistical analysis

All outcomes were dichotomous, and the results were expressed for each trial as an odds ratio (OR) with a 95% confidence interval (CI). Multiple live births (for example twins or triplets) were counted as one live birth event^3^. To examine the associations between AH and interested outcomes, the summary OR with 95% CIs were estimated by summarizing the risk estimates of each study using the random effect models^59^. Heterogeneity between the results of different trials was examined using the *I*^2^ statistic. Statistical heterogeneity was deemed significant if the *P* value was ≤0.1; that is, an indication of more variation than would be expected by chance. *I*^2^ values were also examined and high values (>50%) were taken to indicate substantial heterogeneity.

To investigate the possible sources of heterogeneity of the main results, we carried out stratified analyses by the following study features: hatching method (chemical, laser or mechanical); conception mode (intracytoplasmic sperm injection (ICSI) only, *in vitro* fertilization (IVF) only, and either or unmentioned); number of participants in the AH group (<100 versus ≥100); the extent of AH (thinning only, breach by hole only, complete removal of zona or expansion of zona); embryo transfer status (fresh embryos versus frozen-thawed embryos or unknown); with previous failure history (yes versus no); embryo transfer status with previous failure history (fresh embryos without failure history, fresh embryos with failure history, and frozen-thawed embryos without failure history).

Small study bias, such as publication bias, was evaluated with Egger's regression asymmetry test^60^ and Begg's rank-correlation test^61^. A *P*-value of 0.05 was used to determine whether significant publication bias existed. Additionally, sensitivity analyses were conducted by deleting each study in turn to reflect the influence of individual data on the overall estimate. All statistical analyses were performed with Stata (version 12; StataCorp, College Station, TX).

## Additional Information

**How to cite this article**: Li, D. *et al*. Effect of assisted hatching on pregnancy outcomes: a systematic review and meta-analysis of randomized controlled trials. *Sci. Rep.* **6**, 31228; doi: 10.1038/srep31228 (2016).

## Supplementary Material

Supplementary Information

## Figures and Tables

**Table 1:** Characteristics of the included studies.

First author (ref.), year, Country	Age of intervention/control (Mean ± SD)	Conception mode	AH method	Embryos transfer status	Participants with previous failure history
Wan^13^, 2014, China	33.1 ± 3.7/32.6 ± 3.4	IVF/ICSI	Laser	Fresh	Yes
Razi^29^, 2013, Iran	30.9 ± 0.5/31.6 ± 0.4	ICSI	Laser	Fresh	No
Fang^14^, 2010, China	32.3 ± 3.4/32.1 ± 2.6	IVF/ICSI	Mechanical	Frozen-thawed	Yes
Hagemann^5^, 2010, USA	32.1 ± 3.0/31.2 ± 3.5	IVF	Chemical	Fresh	Yes
Kutlu^18^, 2010, Turkey	29.9 ± 2.9/28.9 ± 3.4	N/A	Laser	Fresh	No
Valojerdi^30^, 2010, Iran	30.9 ± 5.8/29.9 ± 5.1	N/A	Laser	Fresh/Frozen-thawed	Yes
Balakier^33^, 2009, Canada	32.5 ± 3.8/33.8 ± 3.2	IVF	Laser	Fresh	No
Ge^15^, 2008, China	31.1 ± 4.7/30.4 ± 4.2	IVF	Laser	Fresh/Frozen-thawed	No
Sagoskin^6^, 2007, USA	34.0 ± 3.3/34.0 ± 3.2	IVF/ICSI	Laser	Fresh	Yes
Balaban^19^, 2006, Turkey	32.4 ± 3.3/32.7 ± 3.1	ICSI	Laser	Frozen-thawed	No
Nadir^34^, 2005, German	33.1 ± 4.2/34.0 ± 3.7	N/A	Laser	Fresh	No
Elhelw^35^, 2005, Egypt	N/A	ICSI	Laser	Fresh	Yes
Ng^16^, 2005, Hong Kong, China	35.0 ± N/A/35.0 ± N/A	N/A	Laser	Frozen-thawed	Yes
Petersen^23^, 2005, Brazil	34.6 ± 4.6/34.1 ± 5.3	ICSI	Laser	Fresh	Yes
Primi^36^, 2004, Switzerland	N/A	IVF	Laser	Fresh	No
Rufas-Sapir^31^, 2004, Israel	N/A	IVF	Chemical	Fresh	Yes
Carter^12^, 2003, USA	N/A	IVF	Laser	Fresh	Yes
Jelinkova^37^, 2003, Czech	32.3 ± 4.2/32.1 ± 3.2	IVF	Chemical	Fresh	Yes
Petersen^24^, 2002, Brazil	N/A	ICSI	Laser	Fresh	Yes
Urman^20^, 2002, Turkey	31.8/31.5	ICSI	Chemical	Fresh	No
Baruffi^25^, 2000, Brazil	31.8 ± 3.6/31.4 ± 3.6	ICSI	Laser	Fresh	No
Isik^21^, 2000, Turkey	29.1 ± 3.6/30.5 ± 5.2	ICSI	Chemical	Fresh	No
Antinori^28^, 1999, Italy	N/A	IVF	Laser	Fresh	Yes
Isiklar^22^, 1999, Turkey	N/A	IVF	Mechanical	Fresh	No
Laffoon^26^, 1999, Italy	N/A	IVF	Mechanical	Fresh	No
Nagy^27^, 1999, Italy	N/A	IVF/ICSI	Laser	Frozen-thawed	No
Hurst^7^, 1998, USA	30 ± 0.9/30 ± 0.8	IVF	Chemical	Fresh	No
Lanzendorf^9^, 1998, USA	38.0 ± 2.0/38.5 ± 1.8	IVF/ICSI	Chemical	Fresh	No
Utsunomiya^38^, 1998, Japan	N/A	IVF/ICSI	Chemical	Fresh	Yes
Chao^17^, 1997, Taipei, China	36.5 ± 5.2/34.0 ± 3.9	IVF	Mechanical	Fresh	Yes
Ryan^39^, 1997, Australia	N/A	N/A	Chemical	Fresh	No
Hellebaut^40^, 1996, Belgium	30.9 ± 4.3/30.8 ± 3.9	IVF/ICSI	Mechanical	Fresh	No
Tucker^10^, 1996, USA	N/A	ICSI	Chemical	Fresh	No
Stein^32^, 1995, Israel	N/A	IVF	Mechanical	Fresh	Yes
Tucker^8^, 1993, USA	34.1 ± 4.8/34.2 ± 4.1	IVF	Chemical	Fresh	No
Cohen^11^, 1992, USA	N/A	N/A	Chemical	Fresh	No

Abbreviations: AH, assisted hatching; IVF, *in vitro* fertilization; ICSI, intracytoplasmic sperm injection; N/A, not available; SD, standard deviation.

**Table 2:** Summary odd ratios for clinical pregnancy in women who underwent assisted hatching compared with those in the control group.

	No. of study	Summary OR (95% CI)	*I*^2^ value (%)	*P*_h_*
Overall	36	1.16 (1.00–1.36)	48.3	<0.01
Hatching method				
Chemical	12	1.26 (1.01–1.57)	17.0	0.28
Laser	18	1.03 (0.81–1.30)	60.0	<0.01
Mechanical	6	1.68 (1.17–2.42)	0	0.44
Conception mode				
ICSI only	9	1.34 (1.03–1.75)	15.1	0.31
IVF only	14	1.12 (0.88–1.44)	45.0	0.04
Either or unmentioned	13	1.13 (0.83–1.55)	62.4	<0.01
No. of participants in AH group				
≥100	13	1.16 (0.94–1.44)	61.5	<0.01
<100	23	1.16 (0.90–1.49)	39.6	0.03
Extent of AH				
Thinning only	13	1.01 (0.77–1.31)	51.2	0.02
Breach by hole only	12	1.10 (0.83–1.45)	48.9	0.03
Complete removal of zona	10	1.50 (1.07–2.10)	39.1	0.10
Expansion of zona	1	1.50 (0.90–2.49)	N/A	N/A
Embryos transfer status				
Fresh embryos	29	1.12 (0.94–1.33)	46.9	<0.01
Frozen-thawed embryos or unknown	8	1.45 (0.96–2.18)	52.5	0.04
With previous failure history				
Yes or unknown	16	1.21 (0.89–1.64)	60.0	<0.01
No	21	1.18 (0.98–1.40)	33.9	0.07
Embryos transfer status and with previous failure history				
Fresh embryos without failure history	18	1.11 (0.92–1.33)	28.7	0.12
Fresh embryos with failure history	9	1.39 (1.01–1.90)	35.4	0.14
Frozen-thawed embryos without failure history	4	1.75 (1.22–2.52)	16.7	0.31

Abbreviations: OR, odds ratio; CI, confidence interval; AH, assisted hatching; IVF, *in vitro* fertilization; ICSI, intracytoplasmic sperm injection; N/A, not available.

^*^P-value for heterogeneity within each subgroup.

**Table 3:** Summary odd ratios for live birth in women who underwent assisted hatching compared with those in the control group.

	No. of study	Summary OR (95% CI)	*I*^2^ value (%)	*P*_h_*
Overall	15	1.09 (0.92–1.30)	0	0.49
Hatching method				
Chemical	9	1.06 (0.85–1.33)	9.0	0.36
Laser	5	1.19 (0.77–1.83)	9.4	0.35
Mechanical	1	1.08 (0.51–2.29)	N/A	N/A
Conception mode				
ICSI only	5	0.81 (0.52–1.26)	36.4	0.18
IVF only	4	1.20 (0.90–1.59)	0	0.55
Either or unmentioned	6	1.42 (0.85–2.37)	0	0.84
No. of participants in AH group				
≥100	3	1.12 (0.89–1.40)	0	0.56
<100	12	1.05 (0.79–1.39)	10.1	0.35
Extent of AH				
Thinning only	4	1.05 (0.83–1.34)	0	0.53
Breach by hole only	8	1.14 (0.83–1.55)	19.6	0.27
Complete removal of zona	3	1.02 (0.56–1.87)	9.0	0.33
Expansion of zona	0	N/A	N/A	N/A
Embryos transfer status				
Fresh embryos	14	1.11 (0.93–1.32)	0	0.47
Frozen-thawed embryos or unknown	2	1.20 (0.51–2.83)	57.2	0.13
With previous failure history				
Yes or unknown	4	1.30 (0.90–1.87)	0	0.59
No	10	1.02 (0.80–1.30)	13.4	0.32
Embryos transfer status and with previous failure history				
Fresh embryos without failure history	9	1.04 (0.80–1.36)	18.1	0.28
Fresh embryos with failure history	1	3.08 (0.75–12.61)	N/A	N/A
Frozen-thawed embryos without failure history	2	1.20 (0.51–2.83)	57.2	0.13

Abbreviations: OR, odds ratio; CI, confidence interval; AH, assisted hatching; IVF, *in vitro* fertilization; ICSI, intracytoplasmic sperm injection; N/A, not available.

^*^P-value for heterogeneity within each subgroup.

**Table 4:** Summary odd ratios for multiple pregnancy in women who underwent assisted hatching compared with those in the control group.

	No. of study	Summary OR (95% CI)	*I*^2^ value (%)	*P*_h_*
Overall	20	1.50 (1.11–2.01)	44.0	0.02
Hatching method				
Chemical	11	1.31 (0.86–2.00)	53.7	0.02
Laser	6	1.87 (1.33–2.63)	0	0.68
Mechanical	3	1.94 (0.43–8.69)	64.4	0.06
Conception mode				
ICSI only	9	1.68 (1.07–2.64)	36.3	0.13
IVF only	4	1.91 (0.86–4.25)	51.3	0.10
Either or unmentioned	7	1.14 (0.69–1.89)	49.1	0.07
No. of participants in AH group				
≥100	9	1.50 (0.99–2.25)	66.9	<0.01
<100	11	1.53 (0.98–2.38)	0	0.47
Extent of AH				
Thinning only	5	1.57 (0.75–3.33)	75.2	<0.01
Breach by hole only	10	1.32 (0.95–1.82)	5.4	0.39
Complete removal of zona	4	2.64 (1.02–6.85)	30.9	0.23
Expansion of zona	1	1.53 (0.85–2.76)	N/A	N/A
Embryos transfer status				
Fresh embryos	18	1.52 (1.10–2.10)	46.3	0.02
Frozen-thawed embryos or unknown	4	1.80 (0.90–3.62)	70.5	0.02
With previous failure history				
Yes or unknown	8	1.41 (0.82–2.43)	45.8	0.07
No	13	1.62 (1.12–2.33)	44.4	0.04
Embryos transfer status and with previous failure history				
Fresh embryos without failure history	11	1.38 (1.01–1.90)	24.2	0.21
Fresh embryos with failure history	3	1.92 (0.88–4.20)	0	0.64
Frozen-thawed embryos without failure history	3	2.39 (0.90–6.33)	76.2	0.02

Abbreviations: OR, odds ratio; CI, confidence interval; AH, assisted hatching; IVF, *in vitro* fertilization; ICSI, intracytoplasmic sperm injection; N/A, not available.

^*^P-value for heterogeneity within each subgroup.

**Table 5:** Summary odd ratios for miscarriage in women who underwent assisted hatching compared with those in the control group.

	No. of study	Summary OR (95% CI)	*I*^2^ value (%)	*P*_h_*
Overall	17	1.03 (0.72–1.48)	0	0.94
Hatching method				
Chemical	10	1.01 (0.64–1.59)	0	0.55
Laser	5	1.03 (0.56–1.90)	0	0.99
Mechanical	2	1.58 (0.25–9.88)	0	0.76
Conception mode				
ICSI only	5	0.94 (0.41–2.17)	0	0.57
IVF only	6	0.94 (0.53–1.70)	0	0.62
Either or unmentioned	6	1.16 (0.68–1.97)	0	0.90
No. of participants in AH group				
≥100	5	1.03 (0.63–1.70)	0	0.97
<100	12	1.03 (0.62–1.72)	0	0.72
Extent of AH				
Thinning only	6	1.04 (0.52–2.07)	0	0.54
Breach by hole only	7	1.07 (0.64–1.77)	0	0.65
Complete removal of zona	3	0.94 (0.36–2.46)	0	0.99
Expansion of zona	1	0.98 (0.31–3.12)	N/A	N/A
Embryos transfer status				
Fresh embryos	15	1.00 (0.69–1.47)	0	0.90
Frozen-thawed embryos or unknown	2	1.29 (0.46–3.68)	0	0.58
With previous failure history				
Yes or unknown	6	1.22 (0.68–2.20)	0	0.66
No	12	0.97 (0.64–1.47)	0	0.94
Embryos transfer status and with previous failure history				
Fresh embryos without failure history	9	0.89 (0.56–1.44)	0	0.86
Fresh embryos with failure history	4	1.17 (0.60–2.28)	0	0.74
Frozen-thawed embryos without failure history	2	1.29 (0.46–3.68)	0	0.58

Abbreviations: OR, odds ratio; CI, confidence interval; AH, assisted hatching; IVF, *in vitro* fertilization; ICSI, intracytoplasmic sperm injection; N/A, not available.

^*^P-value for heterogeneity within each subgroup.

## References

[b1] Kissin D. M. . Assisted hatching: trends and pregnancy outcomes, United States, 2000–2010. Fertil Steril 102, 795–801 (2014). 25044084 10.1016/j.fertnstert.2014.06.013 PMC4307791

[b2] Pfeifer S. . Role of assisted hatching in *in vitro* fertilization: a guideline. Fertil Steril 102, 348–351 (2014). 24951365 10.1016/j.fertnstert.2014.05.034

[b3] Carney S. K. . Assisted hatching on assisted conception (*in vitro* fertilisation (IVF) and intracytoplasmic sperm injection (ICSI). Cochrane Database Syst Rev 12, D1894 (2012). 10.1002/14651858.CD001894.pub5 PMC7063386 23235584

[b4] Martins W. P., Rocha I. A., Ferriani R. A. & Nastri C. O. Assisted hatching of human embryos: a systematic review and meta-analysis of randomized controlled trials. Hum Reprod Update 17, 438–453 (2011). 21474527 10.1093/humupd/dmr012

[b5] Hagemann A. R. . A prospective, randomized, double-blinded study of assisted hatching in women younger than 38 years undergoing *in vitro* fertilization. Fertil Steril 93, 586–591 (2010). 19268926 10.1016/j.fertnstert.2009.01.116

[b6] Sagoskin A. W. . Laser assisted hatching in good prognosis patients undergoing *in vitro* fertilization-embryo transfer: a randomized controlled trial. Fertil Steril 87, 283–287 (2007). 17094975 10.1016/j.fertnstert.2006.07.1498

[b7] Hurst B. S., Tucker K. E., Awoniyi C. A. & Schlaff W. D. Assisted hatching does not enhance IVF success in good-prognosis patients. J Assist Reprod Genet 15, 62–64 (1998). 9513842 10.1007/BF02766826 PMC3455419

[b8] Tucker M. J., Luecke N. M., Wiker S. R. & Wright G. Chemical removal of the outside of the zona pellucida of day 3 human embryos has no impact on implantation rate. J Assist Reprod Genet 10, 187–191 (1993). 8400729 10.1007/BF01239219

[b9] Lanzendorf S. E. . A prospective, randomized, double-blind study for the evaluation of assisted hatching in patients with advanced maternal age. Hum Reprod 13, 409–413 (1998). 9557848 10.1093/humrep/13.2.409

[b10] Tucker M. J. . Enhancement of outcome from intracytoplasmic sperm injection: does co-culture or assisted hatching improve implantation rates? Hum Reprod 11, 2434–2437 (1996). 8981127 10.1093/oxfordjournals.humrep.a019131

[b11] Cohen J., Alikani M., Trowbridge J. & Rosenwaks Z. Implantation enhancement by selective assisted hatching using zona drilling of human embryos with poor prognosis. Hum Reprod 7, 685–691 (1992). 1639990 10.1093/oxfordjournals.humrep.a137720

[b12] Carter J. . Preliminary results of a prospective randomized study to assess the value of laser assisted hatching before cleavage stage embryo transfer among good-prognosis *in vitro* Fertilization (IVF) patients. Fertil Steril 803, S94 (2003).

[b13] Wan C. Y. . Laser-assisted hatching improves clinical outcomes of vitrified-warmed blastocysts developed from low-grade cleavage-stage embryos: a prospective randomized study. Reprod Biomed Online 28, 582–589 (2014). 24631166 10.1016/j.rbmo.2014.01.006

[b14] Fang C. . Mechanically expanding the zona pellucida of human frozen thawed embryos: a new method of assisted hatching. Fertil Steril 94, 1302–1307 (2010). 19782973 10.1016/j.fertnstert.2009.08.014

[b15] Ge H. S., Zhou W., Zhang W. & Lin J. J. Impact of assisted hatching on fresh and frozen-thawed embryo transfer cycles: a prospective, randomized study. Reprod Biomed Online 16, 589–596 (2008). 18413070 10.1016/s1472-6483(10)60466-x

[b16] Ng E. H. . A randomized double-blind controlled study of the efficacy of laser-assisted hatching on implantation and pregnancy rates of frozen-thawed embryo transfer at the cleavage stage. Hum Reprod 20, 979–985 (2005). 15665025 10.1093/humrep/deh724

[b17] Chao K. H. . Assisted hatching increases the implantation and pregnancy rate of *in vitro* fertilization (IVF)-embryo transfer (ET), but not that of IVF-tubal ET in patients with repeated IVF failures. Fertil Steril 67, 904–908 (1997). 9130897 10.1016/s0015-0282(97)81404-5

[b18] Kutlu P., Atvar O. & Vanlioglu O. F. Laser assisted zona thinning technique has no beneficial effect on the ART outcomes of two different maternal age groups. J Assist Reprod Genet 27, 457–461 (2010). 20467801 10.1007/s10815-010-9431-6 PMC2941589

[b19] Balaban B., Urman B., Yakin K. & Isiklar A. Laser-assisted hatching increases pregnancy and implantation rates in cryopreserved embryos that were allowed to cleave *in vitro* after thawing: a prospective randomized study. Hum Reprod 21, 2136–2140 (2006). 16613888 10.1093/humrep/del097

[b20] Urman B. . Zona-intact versus zona-free blastocyst transfer: a prospective, randomized study. Fertil Steril 78, 392–396 (2002). 12137879 10.1016/s0015-0282(02)03238-7

[b21] Isik A. Z., Vicdan K., Kaba A. & Dagli G. Comparison of zona manipulated and zona intact blastocyst transfers: a prospective randomized trial. J Assist Reprod Genet 17, 135–139 (2000). 10911572 10.1023/A:1009410020580 PMC3455664

[b22] Isiklar A. . The effect of mechanical assisted hatching on progression of cleavage stage embryos to the blastocyst stage [abstract]. Fertil Steril 72 (3 Suppl 1), S162 (1999).

[b23] Petersen C. G. . Implantation failures: success of assisted hatching with quarter-laser zona thinning. Reprod Biomed Online 10, 224–229 (2005). 15823228 10.1016/s1472-6483(10)60944-3

[b24] Petersen C. G. . Zona thinning with a noncontact diode laser in ICSI embryos from women of advanced age. J Assist Reprod Genet 19, 512–516 (2002). 12484493 10.1023/A:1020907801849 PMC3455341

[b25] Baruffi R. L. . Zona thinning with noncontact diode laser in patients aged <or = 37 years with no previous failure of implantation: a prospective randomized study. J Assist Reprod Genet 17, 557–560 (2000). 11212860 10.1023/A:1026481729632 PMC3455458

[b26] Laffoon I. S. . The effect of assisted hatching on the outcome of assisted reproductive technology cycles in women under 39 years of age [abstract]. Fertil Steril 72 (3 Suppl 1), S243 (1999).

[b27] Nagy Z. P. . Laser-assisted hatching and removal of degenerated blastomere(s) of frozen-thawed embryos improves pregnancy rate [abstract]. Fertil Steril 72 (3 Suppl 1), S4 (1999).

[b28] Antinori S. . Laser-assisted hatching at the extremes of the IVF spectrum: first cycle and after 6 cycles; a randomized prospective trial. Hum Reprod 141, 122–123 (1999).

[b29] Razi M. H., Halvaei I. & Razi Y. Laser assisted zona hatching does not improve live birth rate in patients undergoing their first ICSI cycles. Iran J Reprod Med 11, 1021–1026 (2013). 24639729 PMC3941401

[b30] Valojerdi M. R. . Effect of laser zona thinning on vitrified-warmed embryo transfer at the cleavage stage: a prospective, randomized study. Reprod Biomed Online 20, 234–242 (2010). 20113961 10.1016/j.rbmo.2009.11.002

[b31] Rufas-Sapir O. . Is assisted hatching beneficial in patients with recurrent implantation failures? Clin Exp Obstet Gynecol 31, 110–112 (2004). 15266762

[b32] Stein A. . Assisted hatching by partial zona dissection of human pre-embryos in patients with recurrent implantation failure after *in vitro* fertilization. Fertil Steril 63, 838–841 (1995). 7890071 10.1016/s0015-0282(16)57490-1

[b33] Balakier H. . Laser zona thinning in women aged <or = 37 years: a randomized study. Fertil Steril 91, 1479–1482 (2009). 18793768 10.1016/j.fertnstert.2008.07.1729

[b34] Nadir C. H. . Impact of assisted hatching on ART outcome in women with endometriosis. Hum Reprod 20, 2546–2549 (2005). 15905297 10.1093/humrep/dei064

[b35] Elhelw B., El Sadek M. M. & Al Nomrosy K. M. Laser assisted hatching may enhance implantation and pregnancy rates on cryopreserved-thawed embryos in patients with repeated implantation failures. A prospective randomised study. ESHRE Copenhagen - poster abstract. (2005).

[b36] Primi M. P. . A European multicentre prospective randomized study to assess the use of assisted hatching with a diode laser and the benefit of an immunosuppressive/antibiotic treatment in different patient populations. Hum Reprod 19, 2325–2333 (2004). 15284215 10.1093/humrep/deh430

[b37] Jelinkova L. . Improved implantation rate after chemical removal of the zona pellucida. Fertil Steril 79, 1299–1303 (2003). 12798874 10.1016/s0015-0282(03)00260-7

[b38] Utsunomiya T., Sato M. & Hirotsuru K. Assisted hatching by zona thinning to multiple-failure *in vitro* fertilization patients [abstract]. Fertil Steril 70 (3 Suppl 1), S328 (1998).

[b39] Ryan J. P. . Failure of assisted hatching to increase pregnancy rates following the transfer of fresh or frozen-thawed day 2 human embryos. Hum Reprod 121, P143 (1997).

[b40] Hellebaut S. . Does assisted hatching improve implantation rates after *in vitro* fertilization or intracytoplasmic sperm injection in all patients? A prospective randomized study. J Assist Reprod Genet 13, 19–22 (1996). 8825162 10.1007/BF02068864

[b41] Balaban B., Urman B., Alatas C., Mercan R., Mumcu A. & Isiklar A. A comparison of four different techniques of assisted hatching. Hum Reprod 17, 1239–1243 (2002). 11980745 10.1093/humrep/17.5.1239

[b42] Lanzendorf S. E., Ratts V. S., Moley K. H., Goldstein J. S., Dahan M. H. & Odem R. R. A randomized, prospective study comparing laser-assisted hatching and assisted hatching using acidified medium. Fertil Steril 87, 1450–1457 (2007). 17207801 10.1016/j.fertnstert.2006.11.030

[b43] Nichols J. & Gardner R. L. Effect of damage to the zona pellucida on development of preimplantation embryos in the mouse. Hum Reprod 4, 180–187 (1989). 2918072 10.1093/oxfordjournals.humrep.a136868

[b44] Malter H. E. & Cohen J. Blastocyst formation and hatching *in vitro* following zona drilling of mouse and human embryos. Gamete Res 24, 67–80 (1989). 2591852 10.1002/mrd.1120240110

[b45] Blake D. A., Forsberg A. S., Johansson B. R. & Wikland M. Laser zona pellucida thinning–an alternative approach to assisted hatching. Hum Reprod 16, 1959–1964 (2001). 11527905 10.1093/humrep/16.9.1959

[b46] Antinori S., Selman H. A., Caffa B., Panci C., Dani G. L. & Versaci C. Zona opening of human embryos using a non-contact UV laser for assisted hatching in patients with poor prognosis of pregnancy. Hum Reprod 11, 2488–2492 (1996). 8981141 10.1093/oxfordjournals.humrep.a019145

[b47] Mantoudis E., Podsiadly B. T., Gorgy A., Venkat G. & Craft I. L. A comparison between quarter, partial and total laser assisted hatching in selected infertility patients. Hum Reprod 16, 2182–2186 (2001). 11574513 10.1093/humrep/16.10.2182

[b48] Petersen C. G. . Laser-assisted hatching of cryopreserved-thawed embryos by thinning one quarter of the zona. Reprod Biomed Online 13, 668–675 (2006). 17169177 10.1016/s1472-6483(10)60657-8

[b49] Moher D., Liberati A., Tetzlaff J. & Altman D. G. Preferred reporting items for systematic reviews and meta-analyses: the PRISMA statement. BMJ 339, b2535 (2009). 19622551 10.1136/bmj.b2535 PMC2714657

[b50] Gong T. T., Wu Q. J., Wang Y. L. & Ma X. X. Circulating adiponectin, leptin and adiponectin-leptin ratio and endometrial cancer risk: Evidence from a meta-analysis of epidemiologic studies. Int J Cancer 137, 1967–1978 (2015). 25899043 10.1002/ijc.29561

[b51] Hou R., Wu Q. J., Gong T. T. & Jiang L. Dietary fat and fatty acid intake and epithelial ovarian cancer risk: evidence from epidemiological studies. Oncotarget 6, 43099–43119 (2015). 26515595 10.18632/oncotarget.5525 PMC4767494

[b52] Wei J. . Cigarette smoking during pregnancy and preeclampsia risk: a systematic review and meta-analysis of prospective studies. Oncotarget 6, 43667–43678 (2015). 26498356 10.18632/oncotarget.6190 PMC4791258

[b53] Wu Q. J., Gong T. T. & Wang Y. Z. Dietary fatty acids intake and endometrial cancer risk: a dose-response meta-analysis of epidemiological studies. Oncotarget 6, 36081–36097 (2015). 26462150 10.18632/oncotarget.5555 PMC4742163

[b54] Wu Q. J. . Parity and endometrial cancer risk: a meta-analysis of epidemiological studies. Sci Rep 5, 14243 (2015). 26373341 10.1038/srep14243 PMC4642705

[b55] Wu Q. J. . Statin use and breast cancer survival and risk: a systematic review and meta-analysis. Oncotarget 6, 42988–43004 (2015). 26472026 10.18632/oncotarget.5557 PMC4767486

[b56] Wu Q. J. . Consumption of fruit and vegetables reduces risk of pancreatic cancer: evidence from epidemiological studies. Eur J Cancer Prev 25, 196–205 (2016). 26075658 10.1097/CEJ.0000000000000171

[b57] Luan N. N. . Nonlinear reduction in risk for colorectal cancer by oral contraceptive use: a meta-analysis of epidemiological studies. Cancer Causes Control 26, 65–78 (2015). 25359305 10.1007/s10552-014-0483-2

[b58] Higgins J. P. . The Cochrane Collaboration's tool for assessing risk of bias in randomised trials. BMJ 343, d5928 (2011). 22008217 10.1136/bmj.d5928 PMC3196245

[b59] Higgins J. P. & Thompson S. G. Quantifying heterogeneity in a meta-analysis. Stat Med 21, 1539–1558 (2002). 12111919 10.1002/sim.1186

[b60] Egger M., Davey S. G., Schneider M. & Minder C. Bias in meta-analysis detected by a simple, graphical test. BMJ 315, 629–634 (1997). 9310563 10.1136/bmj.315.7109.629 PMC2127453

[b61] Begg C. B. & Mazumdar M. Operating characteristics of a rank correlation test for publication bias. Biometrics 50, 1088–1101 (1994). 7786990

