# Supplementary material for: Effect of assisted hatching on pregnancy outcomes: a systematic review and meta-analysis of randomized controlled trials
Source: Sci Rep. 2016 Aug 9;6:31228. doi: 10.1038/srep31228 (PMC4977517; doi:10.1038/srep31228)
Supplement: Supplementary Information [file srep31228-s1.doc]

**Effect of assisted hatching on pregnancy outcomes: a systematic review and meta-analysis of randomized controlled trials**

**Da Li^1^, Da-Lei Yang^1^, Jing An^1^, Jiao Jiao^1^, Yi-Ming Zhou^2^, Qi-Jun Wu^3^,*****, Xiu-Xia Wang^1^,***

^1^Center of Reproductive Medicine, Department of Obstetrics and Gynecology, Shengjing Hospital of China Medical University, Shenyang 110004, China

^2^Department of Medicine, Brigham and Women's Hospital, Harvard Institutes of Medicine, Harvard Medical School, Boston, MA 02115, USA

^3^Department of Clinical Epidemiology, Shengjing Hospital of China Medical University, Shenyang 110004, China

***Correspondence to:**

Qi-Jun Wu, Department of Clinical Epidemiology, Shengjing Hospital, China Medical University, Shenyang 110004, China.

Telephone: 86-024-96615-13648/ 86-18940254922. Fax: 86-024-83955092.

E-mail: wuqj@sj-hospital.org

Xiu-Xia Wang, Center of Reproductive Medicine, Department of Obstetrics and Gynecology, Shengjing Hospital, China Medical University, Shenyang 110004, China

E-mail: wangxxsj@sina.cn

**Supplementary Figure S1**. Methodological quality graph: review authors’ judgements about each methodological quality item presented as percentages across all included studies.


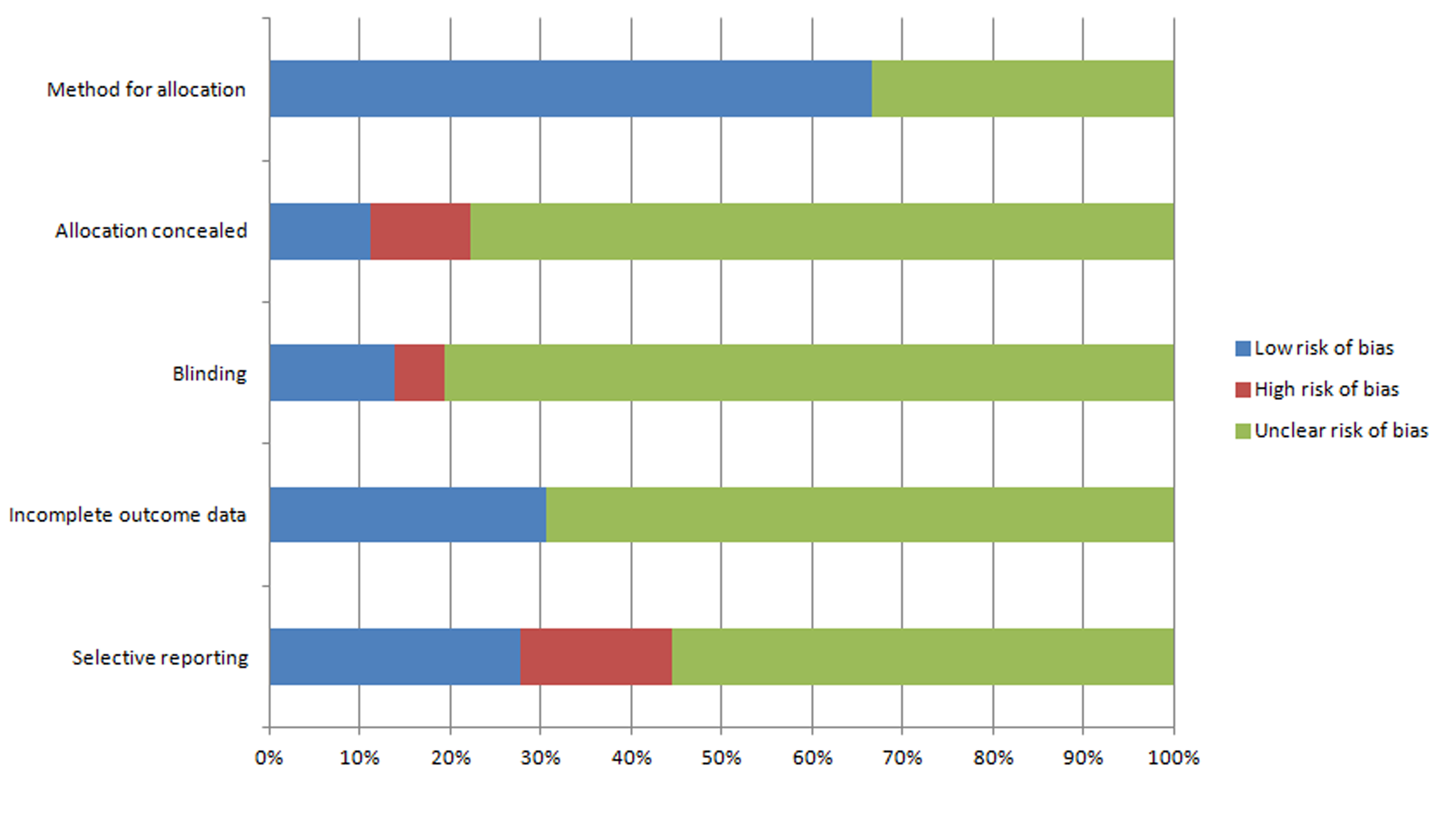


**Supplementary Figure S2**. Forest plots (random effect model) of meta-analysis on the effect of AH on clinical pregnancy.


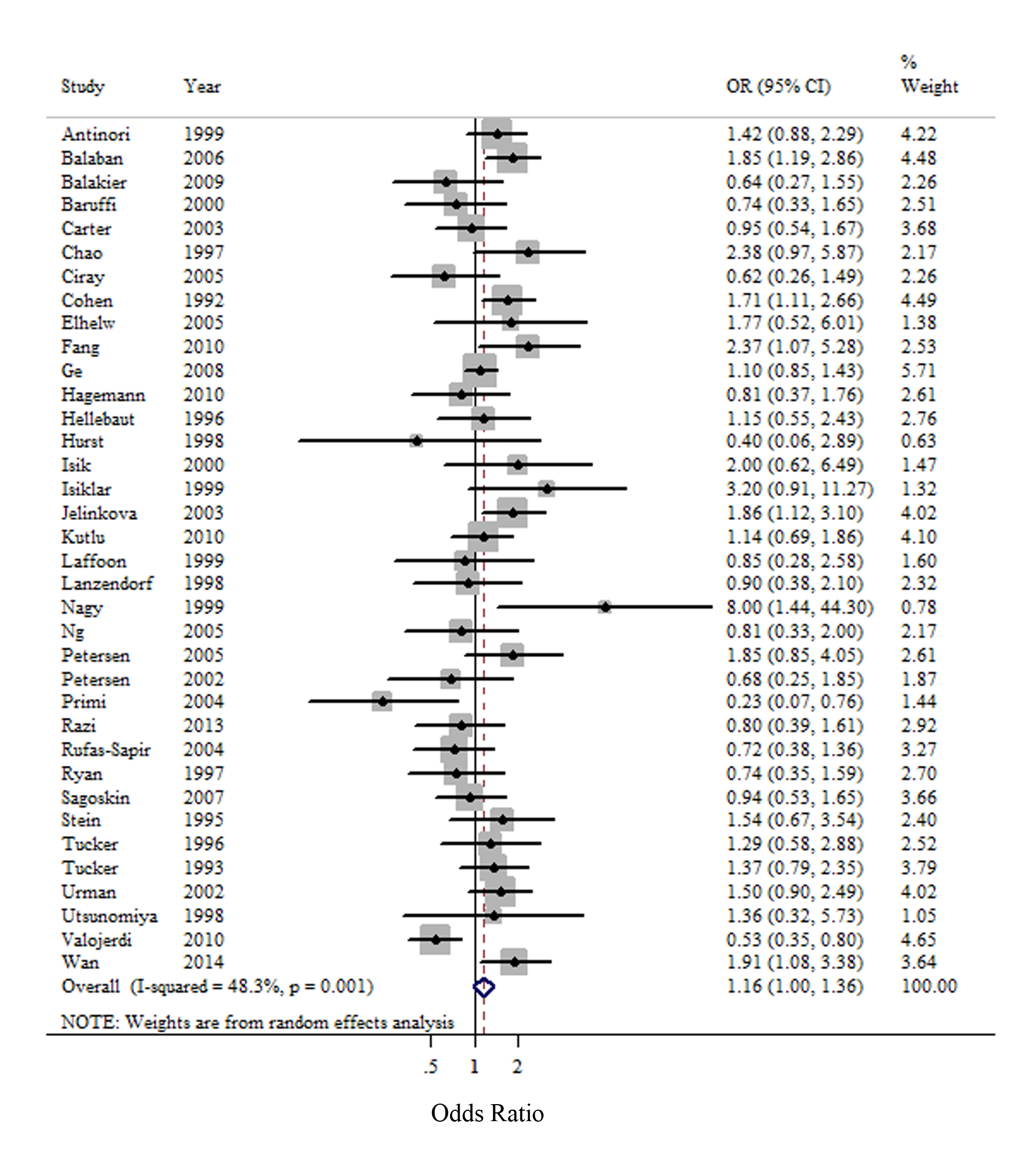


Squares indicate study-specific risk estimates (size of the square reflects the study-specific statistical weight); horizontal lines indicate 95% CIs; diamond indicates the summary relative risk with its 95% CI. OR: odds ratio.

**Supplementary Figure S3**. Forest plots (random effect model) of meta-analysis on the effect of AH on live birth.


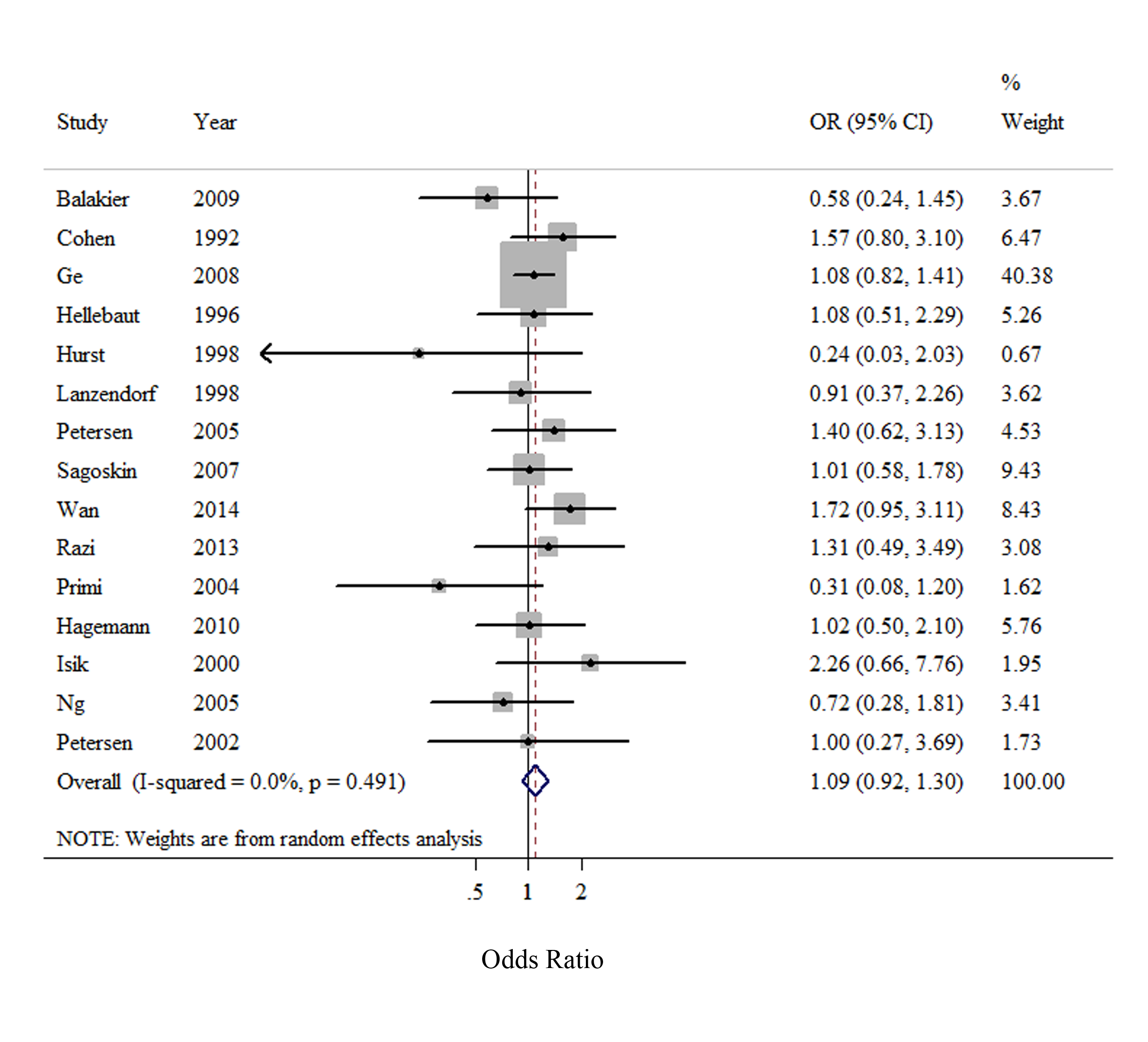


Squares indicate study-specific risk estimates (size of the square reflects the study-specific statistical weight); horizontal lines indicate 95% CIs; diamond indicates the summary relative risk with its 95% CI. OR: odds ratio.

**Supplementary Figure S4**. Forest plots (random effect model) of meta-analysis on the effect of AH on multiple pregnancy.


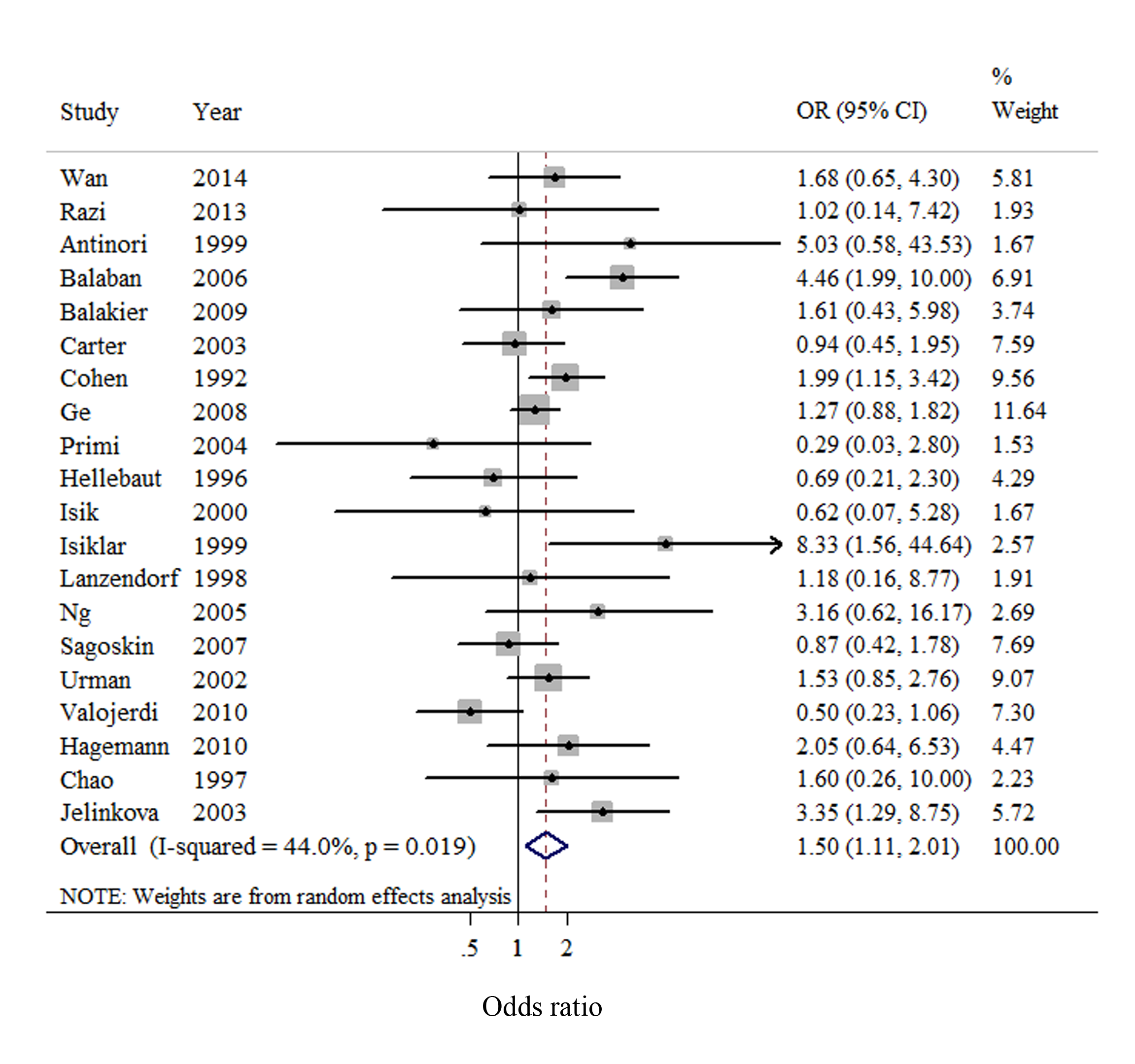


Squares indicate study-specific risk estimates (size of the square reflects the study-specific statistical weight); horizontal lines indicate 95% CIs; diamond indicates the summary relative risk with its 95% CI. OR: odds ratio.

**Supplementary Figure S5**. Forest plots (random effect model) of meta-analysis on the effect of AH on miscarriage.


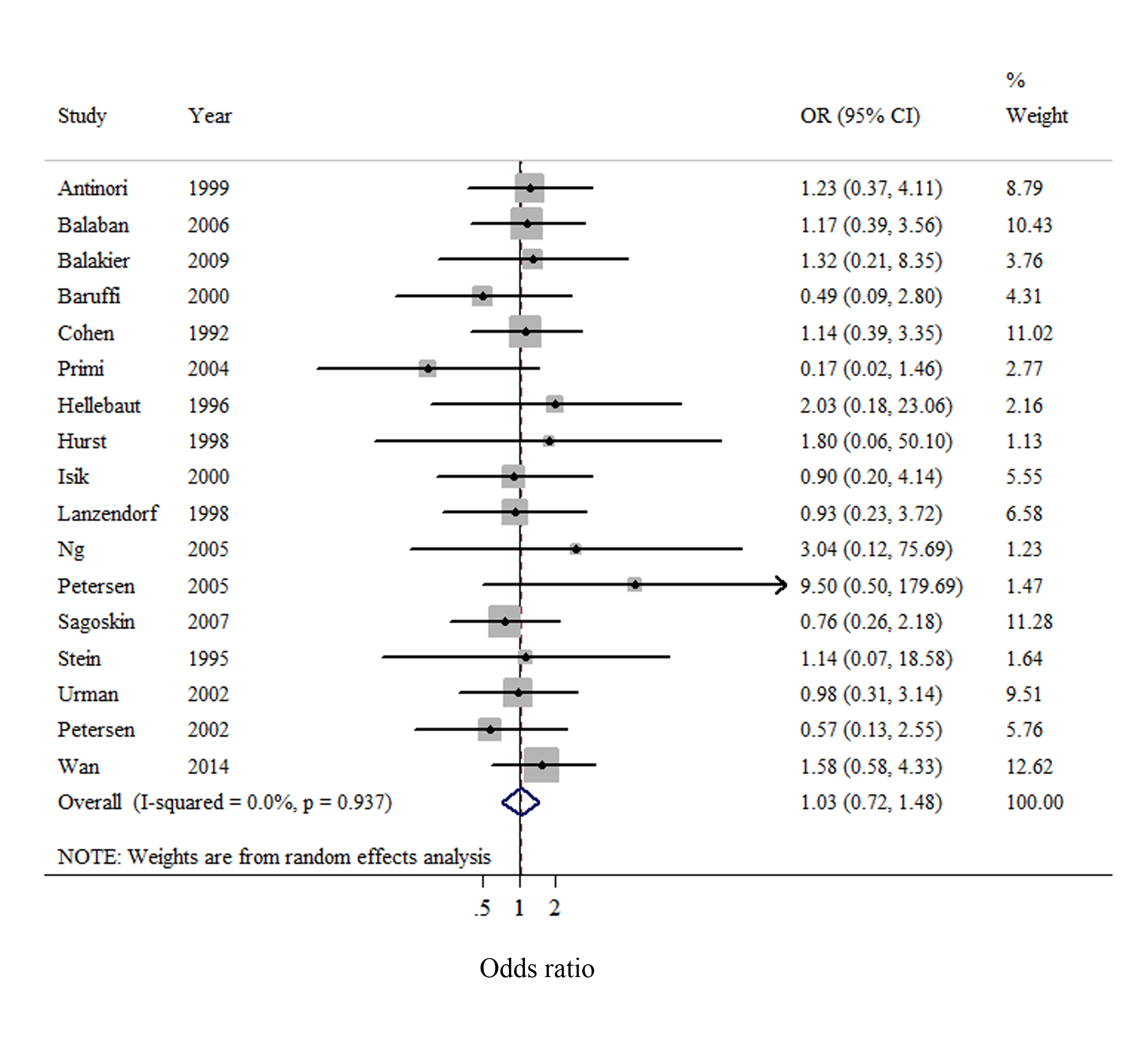


Squares indicate study-specific risk estimates (size of the square reflects the study-specific statistical weight); horizontal lines indicate 95% CIs; diamond indicates the summary relative risk with its 95% CI. OR: odds ratio.

**Supplementary Table S1**. Methodological quality summary: review authors’ judgments about each methodological quality item for each included study.

| **First author (ref), year** | **Method for** **allocation** | **Allocation concealed** | **Blinding** | **Incomplete** **outcome data** | **Selective reporting** |
| --- | --- | --- | --- | --- | --- |
| Wan (13), 2014 | 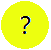 | 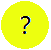 | 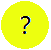 | 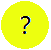 | 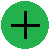 |
| Razi (29), 2013 | 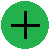 | 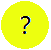 | 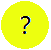 | 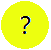 | 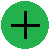 |
| Fang (14), 2010 | 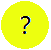 | 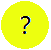 | 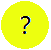 | 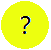 | 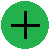 |
| Hagemann (5), 2010 | 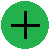 | 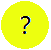 | 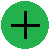 | 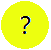 | 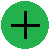 |
| Kutlu (18), 2010 | 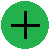 | 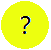 | 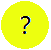 | 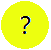 | 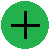 |
| Valojerdi (30), 2010 | 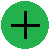 | 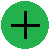 | 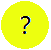 | 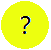 | 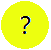 |
| Balakier (33), 2009 | 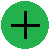 | 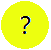 | 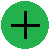 | 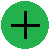 | 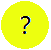 |
| Ge (15), 2008 | 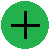 | 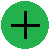 | 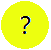 | 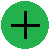 | 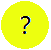 |
| Sagoskin (6), 2007 | 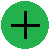 | 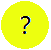 | 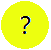 | 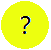 | 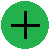 |
| Balaban (19), 2006 | 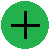 | 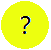 | 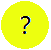 | 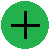 | 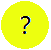 |
| Nadir (34), 2005 | 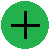 | 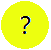 | 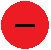 | 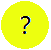 | 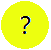 |
| Elhelw (35), 2005 | 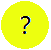 | 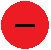 | 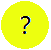 | 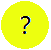 | 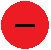 |
| Ng (16), 2005 | 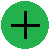 | 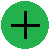 | 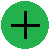 | 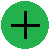 | 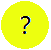 |
| Petersen (23), 2005 | 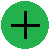 | 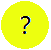 | 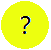 | 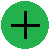 | 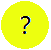 |
| Primi (36), 2004 | 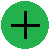 | 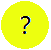 | 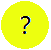 | 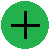 | 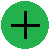 |
| Rufas-Sapir (31), 2004 | 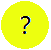 | 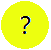 | 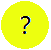 | 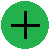 | 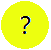 |
| Carter (12), 2003 | 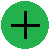 | 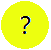 | 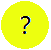 | 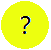 | 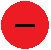 |
| Jelinkova (37), 2003 | 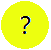 | 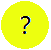 | 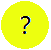 | 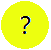 | 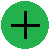 |
| Petersen (24), 2002 | 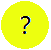 | 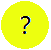 | 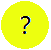 | 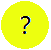 | 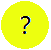 |
| Urman (20), 2002 |  |  |  |  |  |
| Baruffi (25), 2000 |  |  |  |  |  |
| Isik (21), 2000 |  |  |  |  |  |
| Antinori (28), 1999 |  |  |  |  |  |
| Isiklar (22), 1999 |  |  |  |  |  |
| Laffoon (26), 1999 |  |  |  |  |  |
| Nagy (27), 1999 |  |  |  |  |  |
| Hurst (7), 1998 |  |  |  |  |  |
| Lanzendorf (9), 1998 |  |  |  |  |  |
| Utsunomiya (38), 1998 |  |  |  |  |  |
| Chao (17), 1997 |  |  |  |  |  |
| Ryan (39), 1997 |  |  |  |  |  |
| Hellebaut (40), 1996 |  |  |  |  |  |
| Tucker (10), 1996 |  |  |  |  |  |
| Stein (32), 1995 |  |  |  |  |  |
| Tucker (8), 1993 |  |  |  |  |  |
| Cohen (11), 1992 |  |  |  |  |  |

**Supplementary Table S2**. PRISMA Checklist.

| **Section/topic** | **#** | **Checklist item** | **Reported on page #** |
| --- | --- | --- | --- |
| **TITLE** | | |  |
| Title | 1 | Identify the report as a systematic review, meta-analysis, or both. | 1 |
| **ABSTRACT** | | |  |
| Structured summary | 2 | Provide a structured summary including, as applicable: background; objectives; data sources; study eligibility criteria, participants, and interventions; study appraisal and synthesis methods; results; limitations; conclusions and implications of key findings; systematic review registration number. | 2 |
| **INTRODUCTION** | | |  |
| Rationale | 3 | Describe the rationale for the review in the context of what is already known. | 3 |
| Objectives | 4 | Provide an explicit statement of questions being addressed with reference to participants, interventions, comparisons, outcomes, and study design (PICOS). | 3 |
| **METHODS** | | |  |
| Protocol and registration | 5 | Indicate if a review protocol exists, if and where it can be accessed (e.g., Web address), and, if available, provide registration information including registration number. | 9 |
| Eligibility criteria | 6 | Specify study characteristics (e.g., PICOS, length of follow-up) and report characteristics (e.g., years considered, language, publication status) used as criteria for eligibility, giving rationale. | 9 |
| Information sources | 7 | Describe all information sources (e.g., databases with dates of coverage, contact with study authors to identify additional studies) in the search and date last searched. | 9 |
| Search | 8 | Present full electronic search strategy for at least one database, including any limits used, such that it could be repeated. | 9 |
| Study selection | 9 | State the process for selecting studies (i.e., screening, eligibility, included in systematic review, and, if applicable, included in the meta-analysis). | 9-10 |
| Data collection process | 10 | Describe method of data extraction from reports (e.g., piloted forms, independently, in duplicate) and any processes for obtaining and confirming data from investigators. | 10 |
| Data items | 11 | List and define all variables for which data were sought (e.g., PICOS, funding sources) and any assumptions and simplifications made. | 10-11 |
| Risk of bias in individual studies | 12 | Describe methods used for assessing risk of bias of individual studies (including specification of whether this was done at the study or outcome level), and how this information is to be used in any data synthesis. | 10-11 |
| Summary measures | 13 | State the principal summary measures (e.g., risk ratio, difference in means). | 10-11 |
| Synthesis of results | 14 | Describe the methods of handling data and combining results of studies, if done, including measures of consistency (e.g., I^2^) for each meta-analysis. | 10-11 |

| **Section/topic** | **#** | **Checklist item** | **Reported on page #** |
| --- | --- | --- | --- |
| Risk of bias across studies | 15 | Specify any assessment of risk of bias that may affect the cumulative evidence (e.g., publication bias, selective reporting within studies). | 10-11 |
| Additional analyses | 16 | Describe methods of additional analyses (e.g., sensitivity or subgroup analyses, meta-regression), if done, indicating which were pre-specified. | 10-11 |
| **RESULTS** | | |  |
| Study selection | 17 | Give numbers of studies screened, assessed for eligibility, and included in the review, with reasons for exclusions at each stage, ideally with a flow diagram. | 4 |
| Study characteristics | 18 | For each study, present characteristics for which data were extracted (e.g., study size, PICOS, follow-up period) and provide the citations. | 4 |
| Risk of bias within studies | 19 | Present data on risk of bias of each study and, if available, any outcome level assessment (see item 12). | 4, 21 |
| Results of individual studies | 20 | For all outcomes considered (benefits or harms), present, for each study: (a) simple summary data for each intervention group (b) effect estimates and confidence intervals, ideally with a forest plot. | 4-6 |
| Synthesis of results | 21 | Present results of each meta-analysis done, including confidence intervals and measures of consistency. | 4-6 |
| Risk of bias across studies | 22 | Present results of any assessment of risk of bias across studies (see Item 15). | 4-6 |
| Additional analysis | 23 | Give results of additional analyses, if done (e.g., sensitivity or subgroup analyses, meta-regression [see Item 16]). | 4-6 |
| **DISCUSSION** | | |  |
| Summary of evidence | 24 | Summarize the main findings including the strength of evidence for each main outcome; consider their relevance to key groups (e.g., healthcare providers, users, and policy makers). | 7 |
| Limitations | 25 | Discuss limitations at study and outcome level (e.g., risk of bias), and at review-level (e.g., incomplete retrieval of identified research, reporting bias). | 8 |
| Conclusions | 26 | Provide a general interpretation of the results in the context of other evidence, and implications for future research. | 8-9 |
| **FUNDING** | | |  |
| Funding | 27 | Describe sources of funding for the systematic review and other support (e.g., supply of data); role of funders for the systematic review. | 11-12 |

*From:*  Moher D, Liberati A, Tetzlaff J, Altman DG, The PRISMA Group (2009). Preferred Reporting Items for Systematic Reviews and Meta-Analyses: The PRISMA Statement. PLoS Med 6(6): e1000097. doi:10.1371/journal.pmed1000097

For more information, visit: **www.prisma-statement.org**.
